# Functional relationships of three NFU proteins in the biogenesis of chloroplastic iron‐sulfur clusters

**DOI:** 10.1002/pld3.303

**Published:** 2021-02-02

**Authors:** Manasa B. Satyanarayan, Jun Zhao, Jessica Zhang, Fei Yu, Yan Lu

**Affiliations:** ^1^ Department of Biological Sciences Western Michigan University Kalamazoo MI USA; ^2^ State Key Laboratory of Crop Stress Biology for Arid Areas and College of Life Sciences Northwest A&F University Yangling China; ^3^ Present address: Charles River Laboratories Mattawan MI USA; ^4^ Present address: State Key Laboratory of Crop Stress Biology for Arid Areas and College of Life Sciences Northwest A&F University Yangling China

**Keywords:** *Arabidopsis thaliana*, chloroplast, iron‐sulfur clusters, photosynthesis, photosystem I

## Abstract

Iron‐sulfur clusters are required in a variety of biological processes. Biogenesis of iron‐sulfur clusters includes assembly of iron‐sulfur clusters on scaffold complexes and transfer of iron‐sulfur clusters to recipient apoproteins by iron‐sulfur carriers, such as nitrogen‐fixation‐subunit‐U (NFU)‐type proteins. *Arabidopsis thaliana* has three plastid‐targeted NFUs: NFU1, NFU2, and NFU3. We previously discovered that *nfu2* ^−/−^ *nfu3* ^−/−^ mutants are embryo lethal. The lack of viable *nfu2* ^−/−^ *nfu3* ^−/−^ mutants posed a serious challenge. To overcome this problem, we characterized *nfu2‐1* ^−/−^ *nfu3‐2^+/‐^* and *nfu2‐1^+/‐^ nfu3‐2* ^−/−^ sesquimutants. Simultaneous loss‐of‐function mutations in *NFU2* and *NFU3* have an additive effect on the declines of 4Fe‐4S‐containing PSI core subunits. Consequently, the sesquimutants had much lower PSI and PSII activities, much less chlorophyll, and much smaller plant sizes, than *nfu2‐1* and *nfu3‐2* single mutants. These observations are consistent with proposed roles of NFU3 and NFU2 in the biogenesis of chloroplastic 4Fe‐4S. By performing spectroscopic and in vitro reconstitution experiments, we found that NFU1 may act as a carrier for chloroplastic 4Fe‐4S and 3Fe‐4S clusters. In line with this hypothesis, loss‐of‐function mutations in *NFU1* resulted in significant declines in 4Fe‐4S‐ and 3Fe‐4S‐containing chloroplastic proteins. The declines of PSI activity and 4Fe‐4S‐containing PSI core subunits in *nfu1* mutants indicate that PSI is the main target of NFU1 action. The reductions in 4Fe‐4S‐containing PSI core proteins and PSI activity in *nfu3‐2*, *nfu2‐1*, and *nfu1* single mutants suggest that all three plastid‐targeted NFU proteins contribute to the biogenesis of chloroplastic 4Fe‐4S clusters. Although different insertion sites of T‐DNA lines may cause variations in phenotypic results, mutation severity could be an indicator of the relative importance of the gene product. Our results are consistent with the hypothesis that NFU3 contributes more than NFU2 and NFU2 contributes more than NFU1 to the production of 4Fe‐4S‐containing PSI core subunits.

## 1 INTRODUCTION

Iron‐sulfur clusters are essential to all living organisms, because they participate in a variety of biological processes, such as oxidation‐reduction reactions, photosynthesis, respiration, and DNA metabolism (Kohbushi et al., 2009). Due to the varying redox potential of iron, iron‐sulfur clusters have the ability to transfer electrons (Beinert, 2000). In higher plants, iron‐sulfur clusters are best known for participating in photosynthetic electron transport in the chloroplast thylakoid membranes and respiratory electron transport in the inner mitochondrial membrane (Balk & Lobréaux, 2005; Balk & Pilon, 2011; Balk & Schaedler, 2014; Couturier et al., 2013; Johnson et al., 2005; Pilon et al., 2006). Common types of iron‐sulfur clusters found in chloroplasts include 2Fe‐2S, 3Fe‐4S, and 4Fe‐4S (Lu, 2018). Examples of chloroplastic iron‐sulfur proteins include 2Fe‐2S‐containing chloroplast ferredoxin (cFD; Hanke & Mulo, 2013; Hase et al., 2006); 3Fe‐4S‐containing ferredoxin‐glutamine oxoglutarate aminotransferase (FD‐GOGAT; Coschigano et al., 1998); and 4Fe‐4S‐containing Photosystem I core proteins PsaA, PsaB, and PsaC (Psa stands for Photosystem I; Amann et al., 2004; Golbeck, 2003; Lezhneva et al., 2004; Saenger et al., 2002; Schwenkert et al., 2010; Vassiliev et al., 1998).

The biogenesis of chloroplastic iron‐sulfur clusters is a two‐step process (Balk & Pilon, 2011; Couturier et al., 2013; Lill, 2009; Lu, 2018; Przybyla‐Toscano et al., 2018). The first step is the assembly of iron‐sulfur clusters on a scaffold complex by the sequential action of cysteine desulfurase and sulfur transferase (Léon et al., 2002, 2005; Singh et al., 2013; Turowski et al., 2012). The second step is the transfer of newly assembled iron‐sulfur clusters from the scaffold complex to recipient apoproteins by iron‐sulfur carriers, such as nitrogen‐fixation‐subunit‐U (NFU)‐type proteins (Léon et al., 2003). NFU‐type proteins exist ubiquitously in eukaryotes and nitrogen‐fixing prokaryotes (Léon et al., 2003; Lill, 2009). In the higher plant *Arabidopsis thaliana*, there are three nuclear‐encoded plastid‐targeted NFU proteins: NFU1, NFU2, and NFU3 (Léon et al., 2003; Lu, 2018; Przybyla‐Toscano et al., 2018; Yabe et al., 2004). Full‐length NFU1, NFU2, and NFU3 contain a plastid transit peptide, a redox‐active NFU domain with a conserved CXXC motif (C stands for cysteine, X stands for any amino acid), and a redox‐inactive NFU domain (Léon et al., 2003; Lu, 2018; Przybyla‐Toscano et al., 2018; Yabe et al., 2004). The chloroplast localization of NFU1, NFU2, and NFU3 had been demonstrated by fluorescent protein tagging and confocal microscopic analysis (Léon et al., 2003; Nath et al., 2017; Roland et al., 2020).

NFU2 was proposed to participate in the biogenesis of chloroplastic 4Fe‐4S and 2Fe‐2S clusters (Berger et al., 2020; Gao et al., 2013, 2018; Hu et al., 2017; Touraine et al., 2004, 2019; Yabe et al., 2004, 2008). The loss‐of‐function *nfu2‐1* mutant had lower amounts of 4Fe‐4S‐containing PsaA, PsaB, and PsaC and 2Fe‐2S‐containing cFD (Touraine et al., 2004; Yabe et al., 2004). The reduction in the amounts of 4Fe‐4S‐containing PSI core proteins resulted in reduced PSI and PSII activities and a reduced plant size in the *nfu2‐1* mutant (Touraine et al., 2004; Yabe et al., 2004). These observations suggest that 4Fe‐4S‐containing PSI core proteins and 2Fe‐2S‐containing chloroplastic proteins are the main targets of NFU2 action. In line with the hypothesized role of NFU2 in the biogenesis of chloroplastic 2Fe‐2S and 4Fe‐4S clusters (Touraine et al., 2004), the recombinant NFU2 protein was found to accommodate 2Fe‐2S and 4Fe‐4S clusters and is capable of transferring 2Fe‐2S and 4Fe‐4S clusters to recipient apoproteins, such as cFD (Berger et al., 2020; Gao et al., 2013, 2018; Touraine et al., 2019; Yabe et al., 2004). Furthermore, in vitro reconstitution experiments demonstrated that the recombinant NFU2 protein has an iron‐sulfur scaffold function (Gao et al., 2013).

NFU3 was proposed to be involved in the biogenesis of chloroplastic 4Fe‐4S and 3Fe‐4S clusters (Nath et al., 2016, 2017; Touraine et al., 2019). The loss‐of‐function *nfu3‐1* and *nfu3‐2* mutants had reduced amounts of 4Fe‐4S‐containing PSI core proteins PsaA, PsaB, and PsaC (Nath et al., 2016; Touraine et al., 2019). The substantial declines of PSI core subunits in the *nfu3‐1* and *nfu3‐2* mutants resulted in reduced PSI and PSII activities and a reduced plant size (Nath et al., 2016, 2017; Touraine et al., 2019). Consistent with the hypothesized role of NFU3, the recombinant NFU3 protein displayed features of 4Fe‐4S and 3Fe‐4S clusters (Nath et al., 2016). In addition, in vitro reconstitution experiments indicated that the recombinant NFU3 protein has an iron‐sulfur scaffold function (Nath et al., 2016).

In this work, we investigated the functional relationships among three plastid‐targeted NFU proteins in Arabidopsis. It was previously found that double homozygous *nfu2* ^−/−^ *nfu3* ^−/−^ mutants had an embryo‐lethal phenotype (Nath et al., 2016; Touraine et al., 2019). Due to the lack of viable *nfu2* ^−/−^ *nfu3* ^−/−^ mutants, we analyzed the phenotypes of the *nfu2‐1* ^−/−^ *nfu3‐2* ^+/‐^ and *nfu2‐1* ^+/‐^ *nfu3‐2* ^−/−^ sesquimutants in this study. Specifically, we investigated the plant size, the total chlorophyll content, PSI activity, and PSII activity in the sesquimutants and compared to those in the single mutants. Because NFU2 and NFU3 were both proposed to participate in the biogenesis of 4Fe‐4S clusters in the chloroplast, we determined the amounts of 4Fe‐4S‐containing PSI core subunits in the sesquimutants and compared to those in the single mutants. We found that concurrent loss‐of‐function mutations in the *NFU2* and *NFU3* genes had an additive effect on the declines of 4Fe‐4S‐containing PSI core subunits. Unlike NFU2 or NFU3, the function of NFU1 in the biogenesis of chloroplastic iron‐sulfur clusters had been understudied. Therefore, we investigated whether the recombinant NFU1 protein contains 4Fe‐4S and 3Fe‐3S clusters and possesses an iron‐sulfur scaffold function. This was accomplished via spectroscopic analysis and in vitro iron‐sulfur cluster reconstitution experiments of the recombinant NFU1 protein. We also studied whether loss‐of‐function mutations in the *NFU1* gene result in reductions in the plant size, the total chlorophyll content, PSI activity, and PSII activity. To further investigate the function of NFU1, we determined the amounts of representative 4Fe‐4S‐ and 3Fe‐4S‐containing chloroplastic proteins in the *nfu1* mutants. Results from these analyses suggest that NFU1 participates in the biogenesis of chloroplastic iron‐sulfur clusters (e.g., 4Fe‐4S and 3Fe‐4S). The significant declines of 4Fe‐4S‐containing PSI core subunits in the *nfu3‐2*, *nfu2‐1*, and *nfu1* single mutants suggest that all three plastid‐targeted NFU proteins contribute to the biogenesis of 4Fe‐4S clusters in the chloroplast. The relative magnitudes of reductions in the abundances of 4Fe‐4S‐containing PSI core proteins in these single mutants allowed us to hypothesize the relative contributions of NFU3, NFU2, and NFU1 to the biogenesis of chloroplastic 4Fe‐4S clusters.

## 2 MATERIALS AND METHODS

### 2.1 Plant materials and growth conditions

Arabidopsis (*Arabidopsis thaliana*) T‐DNA insertion lines *nfu1‐1*, *nfu1‐2*, *nfu2‐1*, and *nfu3‐2* used in this study were obtained from the Arabidopsis Biological Resource Center (stock numbers GABI_661F04, SALK_038073, SALK_039254, and GABI_791C01, respectively). All four single mutants are in the Columbia ecotype (Alonso et al., 2003; Kleinboelting et al., 2012). The genotypes of the *nfu1‐1*, *nfu1‐2*, *nfu2‐1*, and *nfu3‐2* single mutants and the *nfu2‐1* ^−/−^ *nfu3‐2* ^+/‐^ and *nfu2‐1^+/‐^ nfu3‐2* ^−/−^ sesquimutants were confirmed by PCR, using the Phire Plant Direct PCR kit (Thermo Scientific) and genotyping primers listed in Table S1. Plants were grown in a growth chamber on a 12‐hr‐light/12‐hr‐dark photoperiod. The light intensity was 150 μmol photons m^−2^ s^−1^, the temperature was 20°C, and the relative humidity was 50%.

### 2.2 Quantitative RT‐PCR

Quantitative RT‐PCR (qRT‐PCR) was carried out as described by Clark and Lu (2015). Total RNA was extracted from rosette leaves using the RNeasy plant mini kit (QIAGEN), digested with the RNase‐Free DNase I (QIAGEN), and reverse‐transcribed with random primers and Moloney murine leukemia virus reverse transcriptase (Promega). qPCR was performed on a StepOnePlus Real‐Time PCR System (Thermo Fisher) with Power SYBR Green PCR master mix (Thermo Fisher), and qRT‐PCR primers listed in Table S1. The transcript level of *NFU1* was normalized by the transcript level of *ACT2* (At3g18780).

### 2.3 Measurement of chlorophyll and carotenoid contents

Chlorophyll and carotenoid were extracted with 80% acetone in 2.5 mM HEPES‐KOH, pH7.5 and the amounts (mg) of chlorophyll and carotenoid per gram of fresh tissues were determined on a BioMate 3S spectrophotometer (Thermo Scientific) as described by Wellburn (1994).

### 2.4 Measurements of PSI activity

Measurements of PSI activity (i.e., P700 photooxidation) were performed on dark‐adapted detached leaves as described previously (Nath et al., 2016). The redox state of P700 was determined by monitoring the absorbance change at 830 nm (with an 875‐nm reference beam) on the Dual‐PAM‐100 measuring system (Heinz Walz GmbH). Far‐red light‐induced P700 photooxidation (*ΔA*_830 nm_) is calculated as the absorbance change before and after a 39‐s illumination of saturating far‐red light (720 nm at the maximal light intensity corresponding to level 20 in the Dual‐PAM setting). After reaching a steady‐state level of P700 photooxidation by far‐red light, single‐turnover and multiple‐turnover flash pulses of white saturating light were applied.

### 2.5 Measurement of room temperature chlorophyll fluorescence

Chlorophyll fluorescence parameters were measured on dark‐adapted plants at room temperature with the MAXI Version of the IMAGING‐PAM M‐Series chlorophyll fluorescence system (Heinz Walz GmbH), as described by Lu (2011). The maximum photochemical efficiency of PSII (*F_v_/F_m_*) is calculated using the following equation: *F_v_/F_m_* = (*F*_m_ – *F*_o_)/*F*_m_, where *F*_v_, *F*_m_, and *F*_o_ are variable, maximal, and minimal fluorescence of dark‐adapted leaves, respectively.

### 2.6 Isolation of thylakoid membranes

Thylakoid membranes were isolated as described in Lu et al. (2011) with minor modifications. The entire aerial portion of plants (~2 g) was excised and ground into a fine powder in liquid nitrogen with a mortar and pestle. Freshly made grinding buffer (50 mM HEPES‐KOH, pH7.5, containing 330 mM sorbitol, 2 mM EDTA, 1 mM MgCl_2_, 5 mM ascorbate, 0.05% bovine serum albumin, 10 mM NaF, and 0.25 mg/ml of Pefabloc SC protease inhibitor) was added to the frozen powder (~25 ml/g tissues) and the sample was further homogenized by the repeated swirling of the pestle. The resulting homogenate was filtered through a layer of miracloth (EMD Millipore) and centrifuged at 2,500 g for 4 min at 4°C using a swing‐bucket rotor. The pellet was resuspended and centrifuged in resuspension buffer I (50 mM HEPES‐KOH, pH 7.5, containing 5 mM sorbitol, 10 mM NaF, and 0.25 mg/ml of Pefabloc SC). The resulting thylakoid pellet was resuspended and centrifuged in resuspension buffer II (50 mM HEPES‐KOH, pH 7.5, containing 100 mM sorbitol, 10 mM MgCl2, 10 mM NaF, and 0.25 mg/ml of Pefabloc SC. The final pellet was resuspended in a small volume of resuspension buffer II (~1 ml/2 g starting tissues). The chlorophyll in 20 µl of resuspended thylakoid membranes was extracted with 0.98 ml of 80% acetone in 2.5 mM HEPES‐KOH (pH 7.5) and the amount of chlorophyll was determined on a BioMate 3S spectrophotometer (Thermo Scientific) as described by Wellburn (1994). The remaining suspension was frozen in liquid nitrogen and stored at −80°C for further use.

### 2.7 Extraction of leaf total proteins

Leaf total proteins were extracted as described previously (Hackett et al., 2017). Mature rosette leaves (~50 mg) were excised, frozen in liquid nitrogen, and ground into fine powder with stainless steel beads and TissueLyser II (Qiagen). Freshly made plant protein extraction buffer (50 mM Tris‐HCl, pH 7.5, 150 mM NaCl, 1% Triton X‐100, 0.5% sodium deoxycholate, 0.1% SDS, 1 mM EDTA, 1 mM DTT, and 1% of plant protease inhibitor cocktail) was added to the frozen powder at 5 μl/mg tissues. The samples were further homogenized with TissueLyser II. The resulting homogenates were centrifuged at >10,000 *g* for 3 min at 4°C. The supernatants were transferred to new microfuge tubes and centrifuged again at >10,000 *g* for 3 min at 4°C to remove residual tissue debris. The protein concentrations were determined using the DC (Detergent Compatible) protein assay (Bio‐Rad) with 0 to 1.4 mg/ml of bovine serum albumin as standards.

### 2.8 SDS‐PAGE and immunoblot analysis

SDS‐PAGE and immunoblot analysis of thylakoid membrane proteins was carried out as described previously (Nath et al., 2016). Thylakoid membrane proteins from the wild type, *nfu2* and *nfu3* single mutants and sesquimutants were loaded on an equal fresh tissue weight basis, because the *nfu2* and *nfu3* single mutants and sesquimutants have lower chlorophyll contents than the wild type. Thylakoid membrane proteins from the wild‐type and the *nfu1* single mutants were loaded on an equal chlorophyll basis, because the *nfu1* single mutants have the same amount of chlorophyll as the wild type. Leaf total proteins from the wild‐type and the *nfu1* single mutants were loaded on an equal total protein basis. Proteins were separated with SDS‐PAGE (15% polyacrylamide; 6 M urea), using a Mini PROTEAN® Tetra Cell vertical gel electrophoresis system (Bio‐Rad). After electrophoresis, the proteins were transferred to a polyvinylidene difluoride membrane (EMD Millipore) using the Trans‐Blot electrophoresis transfer cell (Bio‐Rad). The membrane was incubated in the blocking solution (5% nonfat dry milk, 0.1% Tween‐20 in 1X Tris Buffered Saline), then in a diluted primary antibody solution. All the primary antibodies were purchased from Agrisera. Immunodetection of proteins on the polyvinylidene difluoride membrane was performed using the SuperSignal West Pico rabbit IgG detecting kit (Thermo Fisher) and analyzed by the Gel Logic 1500 Imaging System (Kodak).

### 2.9 Expression and purification of the recombinant NFU1 protein

Expression and purification of the recombinant NFU1 protein in *Escherichia coli* were performed as described by Lu et al. (2006) with minor modifications. Total leaf RNA was extracted, digested with RNase‐free DNase I, and reverse‐transcribed with oligo(dT)_15_ primers and Moloney murine leukemia virus reverse transcriptase. Full‐length *NFU1* cDNA (*NFU1* ^1‐696 bp^, corresponding to NFU1^1‐231 AA^) and *NFU1* cDNA lacking the plastid transit peptide (*NFU1* ^208‐696 bp^, corresponding to NFU1^70‐231 AA^) were amplified using the mRNA‐cDNA hybrid, Phusion High‐Fidelity DNA Polymerase (New England Biolabs), forward primers NFU1_BamH1_ATG and NFU1_BamH1_noTP, and a reverse primer NFU1_Xho1_TAG (Table S1). The resulting PCR products were AT‐cloned into the pGEM‐T Easy Vector (Promega) and sequenced to confirm the absence of PCR errors. BamH1/Xho1‐digested *NFU1* fragments were subcloned into the pET28a expression vector (Novagen) and were expressed in *E. coli* strain Rosetta 2 (DE3; Novagen). An overnight culture of Rosetta 2 (DE3) harboring the *NFU1* ^208‐696 bp^ gene was diluted 1:20 and grown at 37°C for 1 hr. Expression of the recombinant NFU1^70‐231 AA^ protein was induced with 1 mM isopropyl β‐D‐thiogalactoside and cells were grown at 28°C overnight. The recombinant NFU1^70‐231 AA^ protein was affinity‐purified with nickel‐nitrilotriacetic acid (Ni‐NTA) agarose under native and aerobic conditions according to the QIAexpressionist protocol (QIAGEN).

### 2.10 Absorption spectra of the as‐purified, reduced, and reconstituted recombinant NFU1 protein

Absorption spectroscopy of the recombinant NFU1^70‐231 AA^ protein was carried out as described previously (Nakamaru‐Ogiso et al., 2002; Schwenkert et al., 2010; Yabe & Nakai, 2006). The absorption spectrum (300–700 nm) of the affinity‐purified recombinant NFU1^70‐231 AA^ protein was recorded on a BioMate 3S spectrophotometer (Thermo Scientific) before and after treating the protein with 10 mM sodium dithionite, a reducing agent capable of reducing iron‐sulfur clusters. In vitro reconstitution of iron‐sulfur clusters on the recombinant NFU1^70‐231 AA^ protein was performed in a Bactron anaerobic chamber as described by Yabe and Nakai (2006). The as‐purified recombinant NFU1^70‐231 AA^ protein was incubated in 100 μM ammonium ferrous sulfate and 100 μM sodium sulfide at 25°C for 2 hr in degassed buffer containing 50 mM Tris‐HCl (pH 7.5), 50 mM NaCl, and 5 mM dithiothreitol. This is followed by a desalting step using an Illustra NAP‐10 column (GE Healthcare Life Sciences) and the absorption spectrum of the reconstituted recombinant NFU1^70‐231 AA^ protein was recorded.

## 3 RESULTS

### 3.1 Phenotypic characterization of the *nfu2‐1* ^−/−^ *nfu3‐2* ^+/‐^ and *nfu2‐1^+/‐^ nfu3‐2* ^−/−^ sesquimutants

**FIGURE 1:**
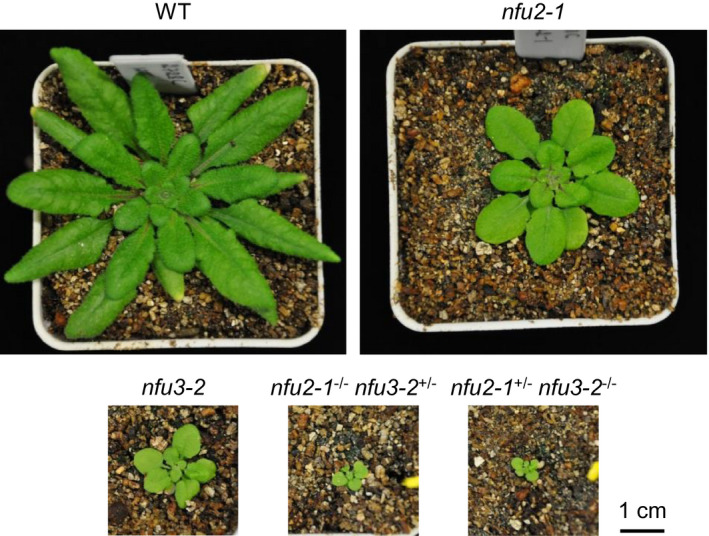
Images of 4‐week‐old wild type, *nfu2* and *nfu3* single mutants and sesquimutants grown on a 12‐hr light/12‐hr dark photoperiod with an irradiance of 150 μmol photons m^−2^ s^−1^ during the light period. WT, wild type. All five plant images are on the same scale

As described previously, double homozygous *nfu2* ^−/−^ *nfu3* ^−/−^ mutants had an embryo‐lethal phenotype (Nath et al., 2016; Touraine et al., 2019). Due to the lack of viable *nfu2* ^−/−^ *nfu3* ^−/−^ mutants, it was challenging to investigate the functional relationship between NFU2 and NFU3. To overcome this problem, we isolated the *nfu2‐1* ^−/−^ *nfu3‐2* ^+/‐^ and *nfu2‐1^+/‐^ nfu3‐2* ^−/−^ sesquimutants. We crossed the homozygous *nfu2‐1* mutant (Touraine et al., 2004; Yabe et al., 2004) with the homozygous *nfu3‐2* mutant (Nath et al., 2016, 2017). The resulting F_1_ population was screened for double heterozygous *nfu2* ^+/‐^ *nfu3* ^+/‐^ mutants. Genotyping was performed by amplifying DNA from 2‐week‐old seedlings, using the Phire Plant Direct PCR kit (Thermo Scientific) and genotyping primers listed in Table S1. The self‐fertilized segregating F_2_ seeds were harvested from double heterozygous *nfu2* ^+/‐^ *nfu3* ^+/‐^ plants and were then sown on the soil. The resulting F_2_ seedlings were genotyped to screen for the *nfu2‐1* ^−/−^ *nfu3‐2* ^+/‐^ and *nfu2‐1^+/‐^ nfu3‐2* ^−/−^ sesquimutants. The genotype of each *nfu2‐1* ^−/−^ *nfu3‐2* ^+/‐^ and *nfu2‐1^+/‐^ nfu3‐2* ^−/−^ sesquimutant plant was carefully confirmed, prior to plant imaging, chlorophyll fluorescence imaging, or tissue harvesting. As shown in Figure 1, the *nfu2‐1* ^−/−^ *nfu3‐2* ^+/‐^ and *nfu2‐1^+/‐^ nfu3‐2* ^−/−^ sesquimutants had a pale green color and were much smaller than the *nfu2‐1* and *nfu3‐2* single mutants at the same age. These data demonstrate that the *nfu2‐1* ^−/−^ *nfu3‐2* ^+/‐^ and *nfu2‐1^+/‐^ nfu3‐2* ^−/−^ sesquimutants suffer further delays in plant growth and development than the *nfu2‐1* and *nfu3‐2* single mutants.

### 3.2 The *nfu2‐1* ^−/−^ *nfu3‐2* ^+/‐^ and *nfu2‐1^+/‐^ nfu3‐2* ^−/−^ sesquimutants displayed lower chlorophyll contents and PSI and PSII activities than the single mutants

**FIGURE 2:**
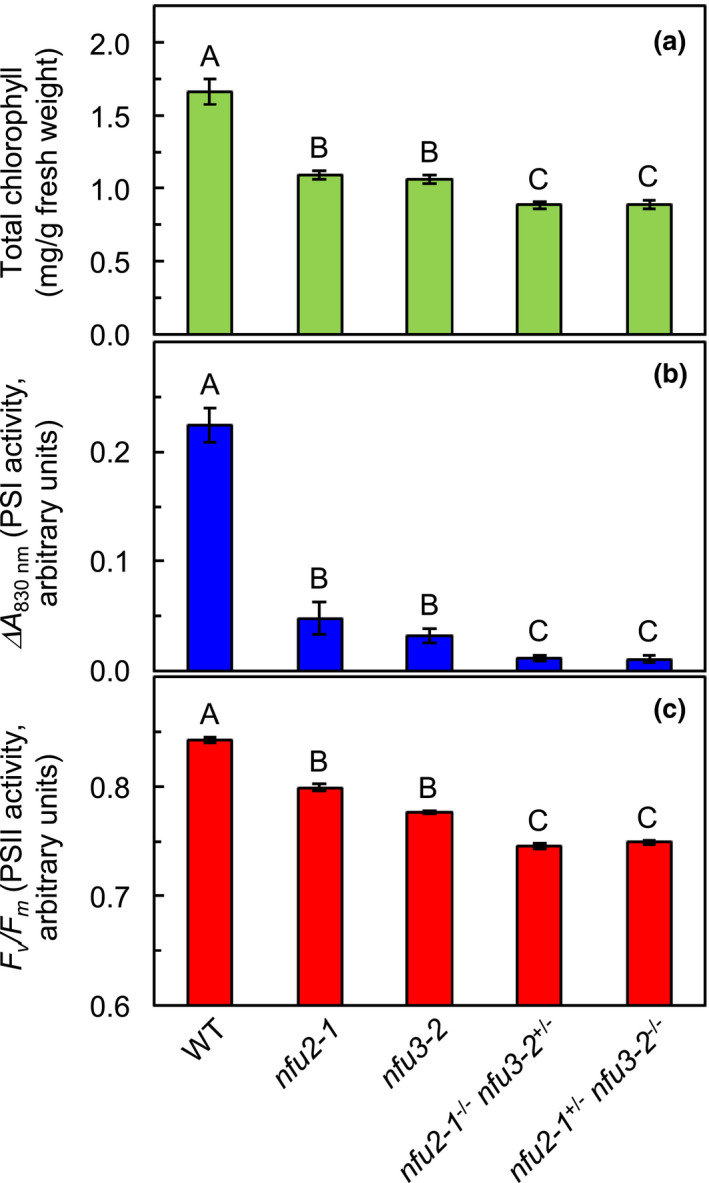
Total chlorophyll content, PSI activity, and PSII activity in the wild type, *nfu2* and *nfu3* single mutants and sesquimutants. (a) Total chlorophyll content. (b) PSI activity. (c) PSII activity. Values not connected by the same upper‐case letter are significantly different (Student's *t* test, *p* < .05). WT, wild type. Note that the Y‐axis in C starts from 0.6 to illustrate the differences between different genotypes. Six‐week‐old plants were used for chlorophyll, PSI activity, and PSII activity measurements in this figure

To inspect whether the *nfu2‐1* ^−/−^ *nfu3‐2* ^+/‐^ and *nfu2‐1^+/‐^ nfu3‐2* ^−/−^ sesquimutants suffer additional decreases in chlorophyll, we determined total chlorophyll contents in 6‐week‐old plants (Figure 2). As shown in Figure 2a, the levels of total chlorophyll in the *nfu2‐1* ^−/−^ *nfu3‐2* ^+/‐^ and *nfu2‐1^+/‐^ nfu3‐2* ^−/−^ sesquimutants were ~47% lower than that in the wild type and ~18% lower than those in the *nfu2‐1* and *nfu3‐2* single mutants.

To investigate the molecular mechanism for the additional growth retardation in the *nfu2‐1* ^−/−^ *nfu3‐2* ^+/‐^ and *nfu2‐1^+/‐^ nfu3‐2* ^−/−^ sesquimutants, we assessed PSI activity in 6‐week‐old plants. This was achieved by measuring far‐red light‐induced photooxidation of P700, where P700 is the PSI reaction‐center chlorophyll *a* molecule whose absorption spectrum peaks at 700 nm (Baker et al., 2007). As shown in Figure 2b, PSI activity in the *nfu2‐1* ^−/−^ *nfu3‐2* ^+/‐^ and *nfu2‐1^+/‐^ nfu3‐2* ^−/−^ sesquimutants was 95% lower than that in the wild type, 77% lower than that in the *nfu2‐1* single mutant, and 66% lower than that in the *nfu3‐2* single mutant. This shows that the *nfu2‐1* ^−/−^ *nfu3‐2* ^+/‐^ and *nfu2‐1^+/‐^ nfu3‐2* ^−/−^ sesquimutants suffer further reductions in PSI activity than the *nfu2‐1* and *nfu3‐2* single mutants.

We also performed chlorophyll fluorescence measurements to determine the activity of PSII in 6‐week‐old plants. As shown in Figure 2c, the maximum photochemical efficiency of PSII (*F*_v_ */F*_m_) in the *nfu2‐1* ^−/−^ *nfu3‐2* ^+/‐^ and *nfu2‐1^+/‐^ nfu3‐2* ^−/−^ sesquimutants was 11% lower than that in the wild type, 6.5% lower than that in the *nfu2‐1* single mutant, and 4% lower than that in the *nfu3‐2* single mutant. This shows that the *nfu2‐1* ^−/−^ *nfu3‐2* ^+/‐^ and *nfu2‐1^+/‐^ nfu3‐2* ^−/−^ sesquimutants suffer additional declines in PSII activity than the *nfu2‐1* and *nfu3‐2* single mutants.

### 3.3 The abundances of PSI core subunits PsaA, PsaB, and PsaC in the *nfu2‐1* ^−/−^ *nfu3‐2* ^+/‐^ and *nfu2‐1^+/‐^ nfu3‐2* ^−/−^ sesquimutants were lower than those in the single mutants

**FIGURE 3:**
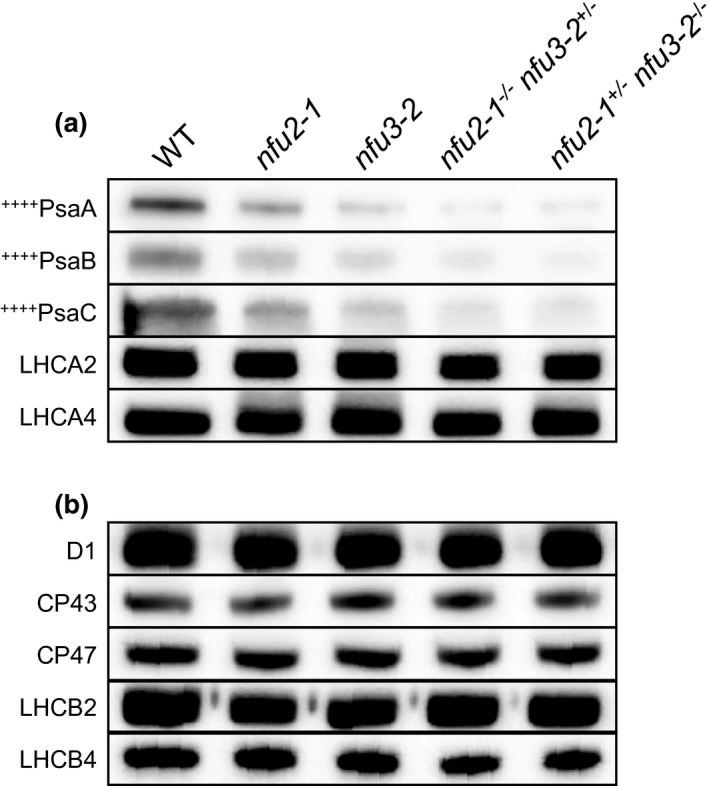
Immunoblots of representative PSI and PSII proteins in the wild type, *nfu2* and *nfu3* single mutants and sesquimutants. (a) Immunoblots of PSI reactive‐center proteins PsaA, PsaB, and PsaC, and PSI‐associated light‐harvesting complex I proteins LHCA2 and LHCA4. (b) Immunoblots of PSII reaction‐center proteins D1, CP43, and CP47 and PSII‐associated light‐harvesting complex II proteins LHCB2 and LHCB4. Thylakoid membrane proteins were used in (a‐b) and were loaded on an equal fresh tissue weight basis. Symbol ++++ indicates that the protein binds to 4Fe‐4S clusters. Five‐week‐old plants were used for SDS‐PAGE and immunoblot analysis in this figure

To investigate why the *nfu2‐1* ^−/−^ *nfu3‐2* ^+/‐^ and *nfu2‐1^+/‐^ nfu3‐2* ^−/−^ sesquimutants displayed additional decreases in the plant size, total chlorophyll content, PSI activity, and PSII activity, we performed SDS‐PAGE and immunoblot analysis on 4Fe‐4S‐containing PSI reaction‐center proteins PsaA, PsaB, and PsaC and other photosynthetic proteins, on an equal fresh tissue weight basis (Figure 3; Table 1). As shown in Figure 3a and Table 1, the average abundance of PSI core subunits PsaA, PsaB, and PsaC in the *nfu2‐1* ^−/−^ *nfu3‐2* ^+/‐^ and *nfu2‐1^+/‐^ nfu3‐2* ^−/−^ sesquimutants was 96% lower than that in the wild type, 82% lower than that in the *nfu2‐1* single mutant, and 67% lower than that in the *nfu3‐2* single mutant. This shows that the *nfu2‐1* ^−/−^ *nfu3‐2* ^+/‐^ and *nfu2‐1^+/‐^ nfu3‐2* ^−/−^ sesquimutants suffer further reductions in the amounts of PSI core subunits than the *nfu2‐1* and *nfu3‐2* single mutants. This observation also suggests that the additional decrease of PSI activity in the *nfu2‐1* ^−/−^ *nfu3‐2* ^+/‐^ and *nfu2‐1^+/‐^ nfu3‐2* ^−/−^ sesquimutants may result from the additional declines of PSI core subunits in the sesquimutants. Unlike PSI core proteins, the abundances of PSI‐associated light‐harvesting complex I proteins LHCA2 and LHCA4 in the *nfu2‐1* ^−/−^ *nfu3‐2* ^+/‐^ and *nfu2‐1^+/‐^ nfu3‐2* ^−/−^ sesquimutants were not substantially lower than those in the *nfu2‐1* and *nfu3‐2* single mutants (Figure 3a; Table 1). Similarly, the amounts of PSII core subunits D1, CP43, and CP47 and PSII‐associated light‐harvesting complex II proteins LHCB2 and LHCB4 in the *nfu2‐1* ^−/−^ *nfu3‐2* ^+/‐^ and *nfu2‐1^+/‐^ nfu3‐2* ^−/−^ sesquimutants were not substantially lower than those in the *nfu2‐1* and *nfu3‐2* single mutants (Figure 3b; Table 1). Therefore, the additional reductions of PSII activity in the *nfu2‐1* ^−/−^ *nfu3‐2* ^+/‐^ and *nfu2‐1^+/‐^ nfu3‐2* ^−/−^ sesquimutants are likely a secondary effect of additional declines in PSI core subunits PsaA, PsaB, and PsaC in the sesquimutants.

**TABLE 1:** Relative abundances of representative PSI and PSII proteins in the wild type, *nfu2* and *nfu3* single mutants and sesquimutants

	WT	*nfu2−1*	*nfu3−2*	*nfu2−1* ^−/−^ *nfu3−2* ^+/‐^	*nfu2−1* ^+/‐^ *nfu3−2* ^−/−^
PsaA^++++^	1.000 ± 0.040^A^	0.286 ± 0.018^B^	0.113 ± 0.010^C^	0.025 ± 0.006^D^	0.023 ± 0.006^D^
PsaB^++++^	1.000 ± 0.041^A^	0.224 ± 0.030^B^	0.124 ± 0.012^C^	0.036 ± 0.005^D^	0.023 ± 0.006^D^
PsaC^++++^	1.000 ± 0.060^A^	0.175 ± 0.032^B^	0.133 ± 0.014^BC^	0.067 ± 0.012^C^	0.069 ± 0.021^BC^
LHCA2	1.000 ± 0.034^A^	0.782 ± 0.023^C^	0.878 ± 0.011^B^	0.661 ± 0.010^D^	0.728 ± 0.032^CD^
LHCA4	1.000 ± 0.120^A^	0.643 ± 0.023^B^	0.710 ± 0.062^B^	0.581 ± 0.034^B^	0.738 ± 0.016^B^
D1	1.000 ± 0.027^A^	0.810 ± 0.033^B^	0.873 ± 0.045^B^	0.826 ± 0.016^B^	0.832 ± 0.023^B^
CP43	1.000 ± 0.020^A^	0.897 ± 0.027^B^	1.013 ± 0.022^A^	0.901 ± 0.011^B^	0.885 ± 0.028^B^
CP47	1.000 ± 0.036^A^	0.926 ± 0.074^AB^	0.960 ± 0.030^AB^	0.843 ± 0.033^BC^	0.780 ± 0.051^C^
LHCB2	1.000 ± 0.062^A^	0.651 ± 0.023^B^	0.749 ± 0.032^B^	0.899 ± 0.020^A^	0.907 ± 0.019^A^
LHCB4	1.000 ± 0.032^A^	0.818 ± 0.001^B^	0.830 ± 0.020^B^	0.650 ± 0.010^C^	0.632 ± 0.045^C^

Note

The values (mean ± SE, *n* = 4) are given as ratios to the protein levels in the wild type (WT). Thylakoid membrane proteins for SDS‐PAGE and immunoblot analysis were loaded on an equal fresh tissue weight basis were used to determine the levels of proteins in this table. Symbols ++++ indicate 4Fe‐4S‐containing proteins. Values not connected by the same letter are significantly different (Student's *t* test, *p* < .05). Five‐week‐old plants were used for SDS‐PAGE and immunoblot analysis in this table.

Taken together, morphological, physiological, and biochemical characterizations of the *nfu2* and *nfu3* single mutants and sesquimutants demonstrate that simultaneous loss‐of‐function mutations in the *NFU2* and *NFU3* genes had additive effects on the abundances of 4Fe‐4S‐containing PSI‐core subunits, PSI activity, PSII activity, chlorophyll content, and plant size, and that NFU2 and NFU3 play major roles in the biogenesis of chloroplastic iron‐sulfur clusters.

### 3.4 The absorption spectrum of recombinant NFU1 showed features characteristic of 4Fe‐4S and 3Fe‐4S clusters

**FIGURE 4:**
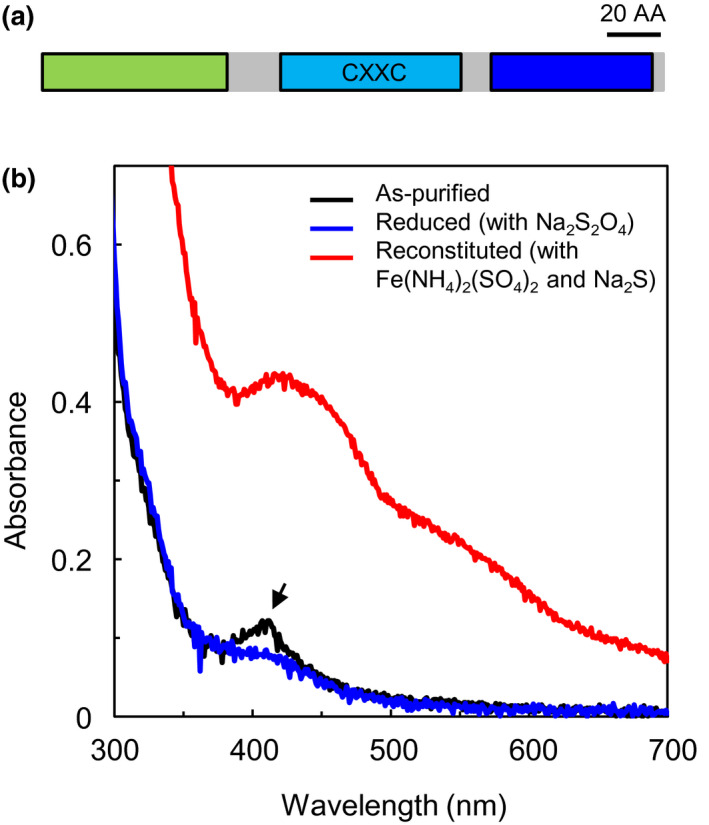
Spectroscopic and reconstitution assays of the recombinant NFU1 protein. (a) Domains in the full‐length NFU1 protein. The green box represents the plastid transit peptide; the cyan box represents the redox‐active NFU domain with the conserved CXXC motif; and the blue box represents the redox‐inactive NFU domain. AA, amino acids. (b) Absorption spectra of the as‐purified, reduced, and reconstituted recombinant NFU1 protein. Recombinant NFU1^70‐231 AA^ was purified aerobically, and an absorption spectrum was recorded (black line). The blue curve represents the absorption spectrum of recombinant NFU1^70‐231 AA^ after reduction with 10 mM sodium dithionite. The black arrow points to the absorption peak at 410 nm, a typical feature of 4Fe‐4S and 3Fe‐4S clusters, which disappears upon reduction by 10 mM sodium dithionite. The red curve represents the absorption spectrum of recombinant NFU1^70‐231 AA^ after reconstitution with ammonium ferrous sulfate and sodium sulfide

The *NFU1* gene is predicted to encode a 231‐amino acid (AA) protein (Figure 4). As described previously, the coding region of NFU1 includes a plastid transit peptide (1‐69 AAs), a redox‐active NFU domain (90‐156 AAs) with the conserved CXXC motif, and a redox‐inactive NFU domain (168‐227 AAs) at the C‐terminus (Figure 4a). Fluorescent protein tagging and confocal microscopic analysis showed that NFU1 is targeted to the chloroplast (Léon et al., 2003; Roland et al., 2020). NFU1 was proposed to bind and deliver 4Fe‐4S clusters and possibly 3Fe‐4S clusters to recipient apoproteins (Roland et al., 2020). Therefore, we expressed the 6xHis‐tagged NFU1 protein in *Escherichia coli* strain Rosetta 2 (DE3) and purified the recombinant NFU1 protein with nickel‐charged agarose resin under native and aerobic conditions. The plastid transit peptide in NFU1 was removed to produce NFU1^70‐231 AA^, to ensure protein solubility. The absorption spectrum of the as‐purified recombinant NFU1^70‐231 AA^ protein had a broad absorption peak around 410 nm (Figure 4b). This 410‐nm broad absorption peak is a feature of 4Fe‐4S and 3Fe‐4S clusters, not a feature of 2Fe‐2S clusters (Kennedy et al., 1984; Nakamaru‐Ogiso et al., 2002). Thus, the absorption spectrum of the recombinant NFU1 protein is consistent with the proposed role of NFU1 in the biogenesis of 4Fe‐4S and 3Fe‐4S clusters.

### 3.5 In vitro reconstitution indicated an iron‐sulfur scaffold function of NFU1

The 410‐nm broad absorption peak of the as‐purified NFU1^70‐231 AA^ protein disappeared after the addition of 1 mM reducing agent sodium dithionite (Figure 4b). This suggests that iron‐sulfur clusters bound to the as‐purified NFU1^70‐231 AA^ protein are redox‐sensitive (Nakamaru‐Ogiso et al., 2002). To test whether NFU1 has an iron‐sulfur scaffold function, we performed in vitro reconstitution of iron‐sulfur clusters on the recombinant NFU1^70‐231 AA^ protein, by applying the protein with an equimolar concentration of ferrous ion and sulfide. The 410‐nm broad absorption peak became more evident after the treatment (Figure 4b). The spectroscopic and in vitro reconstitution experiments demonstrated that NFU1 has an iron‐sulfur scaffold function, especially for 4Fe‐4S and 3Fe‐4S clusters.

### 3.6 Identification and phenotypic characterization of the T‐DNA insertion mutants *nfu1‐1* and *nfu1‐2*

**FIGURE 5:**
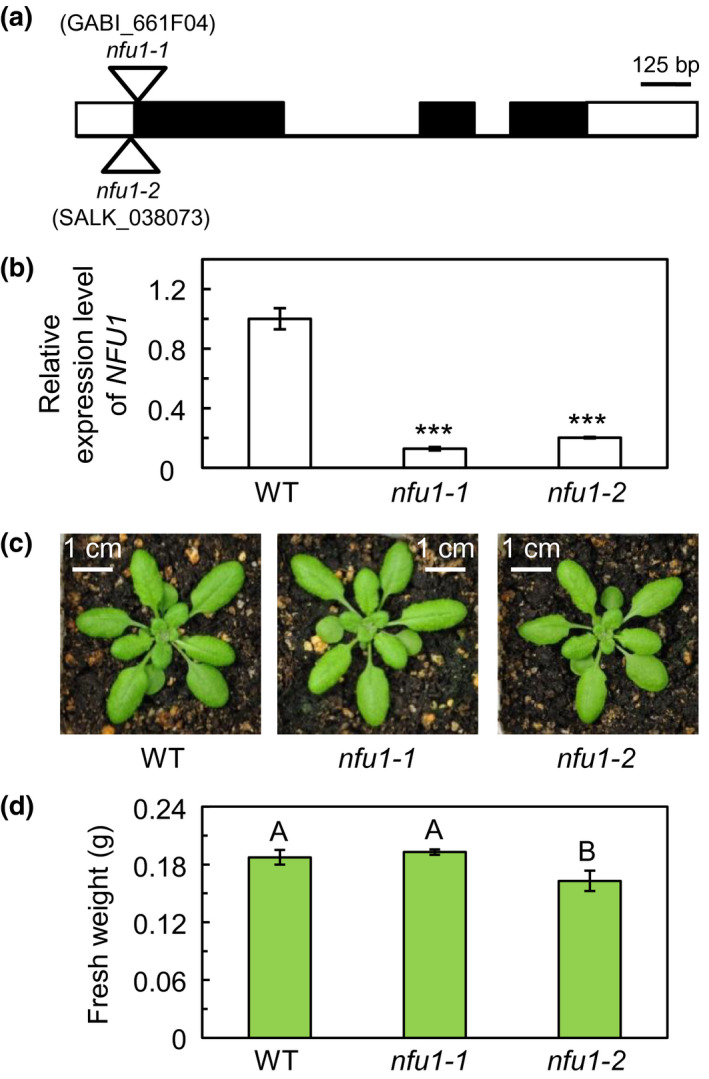
Identification and phenotypic characterization of the *nfu1‐1* and *nfu1‐2* mutants. (a) Structure of the *NFU1* gene and locations of the *nfu1‐1* and *nfu1‐2* mutations. White boxes represent the untranslated regions; black boxes represent exons; and lines represent introns. The T‐DNA insertions in the *nfu1‐1* and *nfu1‐2* mutants are represented by triangles. bp, base pair. (b) Relative expression level of *NFU1* determined by quantitative RT‐PCR in 4‐week‐old plants. The transcript level of *NFU1* was normalized by the transcript level of *ACT2* (At3g18780). The values (mean ± SE, *n* = 5) are given as ratios to the transcript level of *NFU1* in the wild type. Asterisks indicate significant differences between the mutant and the wild type (WT; Student's *t* test; *, *p *<* .05*; **, *p < .01*; ***, *p < .001*). (c) Images of 4‐week‐old plants grown on a 12‐hr light/12‐hr dark photoperiod with an irradiance of 150 μmol photons m^−2^ s^−1^ during the light period. WT, wild type. (d) Fresh weight of the above‐ground portions of 4‐week‐old plants. Values are presented as mean ± SE (*n* = 7). Values not connected by the same upper‐case letter are significantly different (Student's *t* test, *p* < .05)

To study the function of NFU1, we isolated two homozygous T‐DNA insertion mutants of Arabidopsis: *nfu1‐1* (GABI_661F04) and *nfu1‐2* (SALK_038073; Figure 5). The *nfu1‐1* and *nfu1‐2* mutants carry the T‐DNA insertion in the first exon and the 50‐bp 5’‐untranslated region of At4g01940, respectively (Figure 5a). Quantitative reverse transcription (RT)‐PCR showed that the *NFU1* transcript level is substantially decreased in the *nfu1* mutants (Figure 5b). This confirms that *nfu1‐1* and *nfu1‐2* are loss‐of‐function mutants of the *NFU1* gene. The *nfu1‐1* mutant appeared to be phenotypically similar to the wild type (Figure 5c; Touraine et al., 2019). The *nfu1‐2* mutant appeared to be smaller than the wild type (Figure 5c). It is unclear whether the subtle difference in the plant size among the two *nfu1* mutants is due to allelic differences. In line with plant morphology, the aerial portion of the *nfu1‐1* mutant had a similar fresh weight as the wild type, while the aerial portion of *nfu1‐2* was significantly lighter than that of the wild type (Figure 5d).

### 3.7 Pigment contents, PSI activity, and PSII activity in the *nfu1‐1* and *nfu1‐2* mutants

**FIGURE 6:**
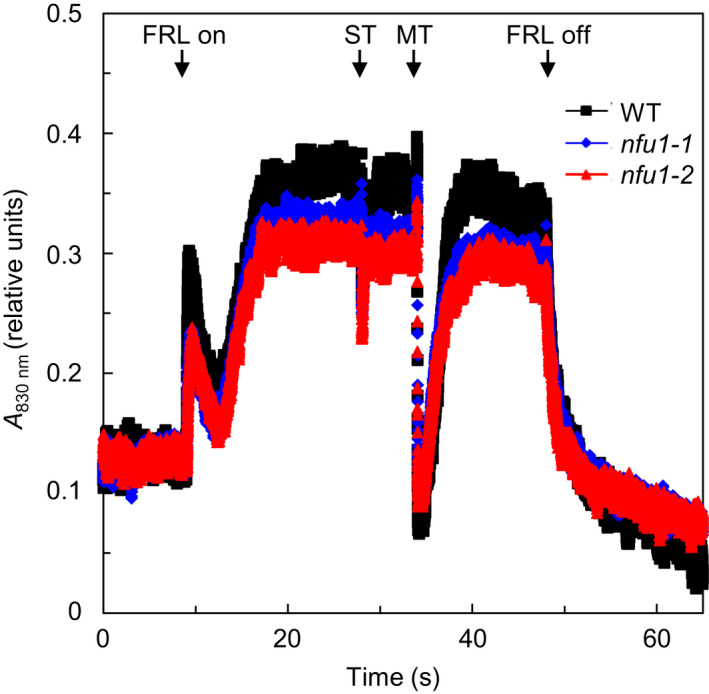
Kinetic measurements of PSI activity (P700 photooxidation) in the wild‐type and *nfu1* mutants. Absorbance of P700 at 830 nm was used as a measurement of the PSI redox state. The measurement was performed on the Dual‐PAM‐100 measuring system (Heinz Waltz GmbH). Far‐red light‐induced P700 photooxidation (*ΔA*_830 nm_) is calculated as the absorbance change before and after a 39‐s illumination of saturating far‐red light (FRL). After reaching a steady‐state level of P700 photooxidation by FRL, single‐turnover (ST) and multiple‐turnover (MT) flash pulses of white saturating light were applied. Representative traces are shown. Four‐week‐old plants were used for PSI activity measurements in this figure

To further investigate the function of NFU1, we measured far‐red light‐induced photooxidation of P700, the PSI reaction‐center chlorophyll *a* molecule whose absorption peaks at 700 nm (Baker et al., 2007). This was achieved by monitoring the absorbance change in P700 at the wavelength of 830 nm, before and after a 39‐s saturating far‐red light illumination (Figure 6; Table 2). The far‐red light‐induced photooxidation of P700 in the *nfu1‐1* and *nfu1‐2* mutants was 10% and 22% lower than that in the wild type, respectively (Table 2), suggesting that loss‐of‐function mutations in the *NFU1* gene cause decrease in PSI activity.

**TABLE 2:** Pigment contents and photosynthetic parameters in the wild‐type and *nfu1* mutants

	WT	*nfu1−1*	*nfu1−2*
*ΔA*_830 nm_ (PSI activity)	0.216 ± 0.009^A^	0.196 ± 0.012^AB^	0.171 ± 0.007^B^
*F*_v_/*F*_m_ (PSII activity)	0.781 ± 0.002^A^	0.782 ± 0.002^A^	0.778 ± 0.003^A^
Total Chl (mg/g FW)	1.267 ± 0.019^A^	1.255 ± 0.019^A^	1.211 ± 0.027^A^
Car (mg/g FW)	0.302 ± 0.005^A^	0.295 ± 0.006^A^	0.287 ± 0.006^A^

Note

Measurements of P700 photooxidation were performed on dark‐adapted, detached leaves, with the Dual‐PAM‐100 measuring system (Heinz Waltz GmbH). Far‐red light‐induced P700 photooxidation (*ΔA*_830 nm_) is calculated as the absorbance change before and after a 39‐s illumination of saturating far‐red light (720 nm). Measurements of chlorophyll fluorescence parameters were performed on dark‐adapted plants, with the IMAGING‐PAM M‐Series chlorophyll fluorescence system (Heinz Waltz GmbH). Chlorophyll and carotenoid were extracted and determined as described by Wellburn (1994). Data are presented as means ± SE (*n* = 6 for P700 photooxidation, *n* = 8 for chlorophyll fluorescence parameters, and *n* = 5 for pigment contents). Values not connected by the same letter are significantly different (Student's *t* test, *p* < .05). Four‐week‐old plants were used for PSI activity, PSII activity, and pigment measurements in this table.

Abbreviations: Chl, chlorophyll; Car, carotenoid; FW, fresh weight.

We also determined the maximum photochemical efficiency of PSII (*F*_v_ */F*_m_), the total chlorophyll content, and the carotenoid content in the *nfu1* mutants and the wild type. Although *F*_v_ */F*_m_, the total chlorophyll content, and the carotenoid content in the *nfu1* mutants were not statistically different from those in the wild type, they followed the same trend as PSI activity (Table 2).

### 3.8 The *nfu1‐1* and *nfu1‐2* mutants had reduced levels of 4Fe‐4S‐ and 3Fe‐4S‐containing chloroplastic proteins

To investigate the molecular mechanism for the reduced PSI activity in the *nfu1* mutants, we extracted thylakoid membrane proteins and performed SDS‐PAGE and immunoblot analysis on several photosynthesis‐related proteins (Table 3; Figure S1). The level of PSI core protein PsaA in the *nfu1‐1* and *nfu1‐2* mutants was 7% and 13% lower than that in the wild type, respectively (Table 3). The average content of PSI core protein PsaC in the *nfu1* mutants was ~ 25% lower than that in the wild type (Table 3). Although the amount of PSI core protein PsaB in the *nfu1* mutants was not statistically different from that in the wild type, it followed the same trend as PsaA and PsaC (Table 3). The relative abundances of these three 4Fe‐4S‐containing PSI core subunits are consistent with the reduced PSI activity in the *nfu1‐1* and *nfu1‐2* mutants (Figure 6; Table 2). Unlike PSI core proteins, the amounts of PSII core subunits D1, CP43, and CP47 in the *nfu1‐1* and *nfu1‐2* mutants were not statistically different from those in the wild type (Table 3). This observation is in line with the lack of PSII‐related phenotype in the *nfu1* mutants (Table 2). We also determined the relative abundances of two cytochrome *b*_6_ *f* complex (Cyt *b*_6_ *f*) proteins PetB and PetC (Photosynthetic electron transfer B and C). PetC is a Rieske iron‐sulfur protein, which contains a Rieske‐type 2Fe‐2S cluster (Hojka et al., 2014). The contents of PetB and PetC in the *nfu1‐1* and *nfu1‐2* mutants were statistically similar to those in the wild type (Table 3). Furthermore, we determined the relative abundances of cFD and FD‐GOGAT, which contain classic 2Fe‐2S and 3Fe‐4S, respectively (Coschigano et al., 1998; Hanke & Mulo, 2013). The level of 2Fe‐2S‐containing cFD in the *nfu1* mutants did not appear to be statistically different from that in the wild type (Table 3). Interestingly, the abundance of 3Fe‐4S‐containing FD‐GOGAT in the *nfu1‐1* and *nfu1‐2* mutants was 10% and 5% lower than that in the wild type (Table 3).

**TABLE 3:** Relative abundances of representative iron‐sulfur cluster‐containing proteins and other photosynthetic proteins in the wild‐type and *nfu1* mutants

	WT	*nfu1−1*	*nfu1−2*
PsaA^++++^	1.00 ± 0.03^A^	0.93 ± 0.02^AB^	0.87 ± 0.02^B^
PsaB^++++^	1.00 ± 0.05^A^	0.87 ± 0.05^A^	0.92 ± 0.03^A^
PsaC^++++^	1.00 ± 0.10^A^	0.74 ± 0.06^B^	0.75 ± 0.04^B^
D1	1.00 ± 0.05^A^	1.12 ± 0.03^A^	1.08 ± 0.04^A^
CP43	1.00 ± 0.06^A^	1.06 ± 0.04^A^	1.10 ± 0.03^A^
CP47	1.00 ± 0.04^A^	1.04 ± 0.00^A^	1.04 ± 0.02^A^
PetB	1.00 ± 0.03^A^	0.94 ± 0.02^A^	0.91 ± 0.02^A^
PetC^††^	1.00 ± 0.10^A^	1.08 ± 0.04^A^	1.05 ± 0.03^A^
cFD^++^	1.00 ± 0.03^A^	0.93 ± 0.02^A^	0.98 ± 0.00^A^
FD‐GOGAT^+++^	1.00 ± 0.02^A^	0.90 ± 0.03^B^	0.95 ± 0.02^AB^

Note

Proteins were immunodetected as in Figure S1. The values (mean ± SE, *n* = 4) are given as ratios to the protein levels in the wild type (WT). Leaf total proteins loaded on an equal total protein basis were used to determine the abundances of cFD and FD‐GOGAT. Because the *nfu1* mutants did not display changes in chlorophyll contents, thylakoid membrane proteins loaded on an equal chlorophyll basis were used to determine the levels of other proteins in this table. Symbols ++, ††, +++, and ++++ indicate that the protein binds classic 2Fe‐2S, Rieske‐type 2Fe‐2S, 3Fe‐4S, and 4Fe‐4S, respectively. Values not connected by the same letter are significantly different (Student's *t* test, *p* < .05). Four‐week‐old plants were used for SDS‐PAGE and immunoblot analysis in this table.

Taken together, spectroscopic and reconstitution analyses of the recombinant NFU1 protein plus morphological, physiological, and biochemical characterizations of the *nfu1* mutants collectively demonstrate that NFU1 also participates in the biogenesis of chloroplastic iron‐sulfur clusters (e.g., 4Fe‐4S and 3Fe‐4S).

## 4 DISCUSSION

The Arabidopsis chloroplast contains three nuclear‐encoded NFU proteins: NFU1, NFU2, and NFU3 (Léon et al., 2003; Lu, 2018; Yabe et al., 2004). As discussed below, we showed that concurrent loss‐of‐function mutations in the *NFU2* and *NFU3* genes had an additive effect on the declines of 4Fe‐4S‐containing PSI core proteins. Consequently, the *nfu2‐1* ^−/−^ *nfu3‐2* ^+/‐^ and *nfu2‐1* ^+/‐^ *nfu3‐2* ^−/−^ sesquimutants had much lower PSI and PSII activities, much less chlorophyll, and a much smaller plant size than the *nfu2‐1* and *nfu3‐2* single mutants. Spectroscopic and reconstitution experiments of the recombinant NFU1 protein, as well as physiological and biochemical characterizations of the loss‐of‐function *nfu1* mutants, suggest that NFU1 may act as a carrier for chloroplastic 4Fe‐4S and 3Fe‐4S clusters. Furthermore, we discussed the relative contributions of NFU3, NFU2, and NFU1 to the biogenesis of chloroplastic 4Fe‐4S clusters, according to the magnitudes of reductions of 4Fe‐4S‐containing PSI core proteins in the corresponding single mutants.

### 4.1 Simultaneous loss‐of‐function mutations in *NFU2* and *NFU3* have additive effects on 4Fe‐4S‐containing PSI core subunits, PSI activity, PSII activity, and mutant phenotypes

As shown in Figure 1, the *nfu2‐1* ^−/−^ *nfu3‐2* ^+/‐^ and *nfu2‐1^+/‐^ nfu3‐2* ^−/−^ sesquimutants were much smaller than the *nfu2‐1* and *nfu3‐2* single mutants at the same age. Consistent with the smaller plant size and paler leaf color, the *nfu2‐1* ^−/−^ *nfu3‐2* ^+/‐^ and *nfu2‐1^+/‐^ nfu3‐2* ^−/−^ sesquimutants had significantly less chlorophyll than the *nfu2‐1* and *nfu3‐2* single mutants (Figure 2a). We also found that PSI and PSII activities in the *nfu2‐1* ^−/−^ *nfu3‐2* ^+/‐^ and *nfu2‐1^+/‐^ nfu3‐2* ^−/−^ sesquimutants were significantly lower than those in the *nfu2‐1* and *nfu3‐2* single mutants (Figure 2b,c). In line with the substantial reductions of PSI activity in the *nfu2‐1* ^−/−^ *nfu3‐2* ^+/‐^ and *nfu2‐1^+/‐^ nfu3‐2* ^−/−^ sesquimutants, the abundances of 4Fe‐4S‐containing PSI core subunits PsaA, PsaB, and PsaC in these sesquimutants were substantially lower than those in the *nfu2‐1* and *nfu3‐2* single mutants (Figure 3a; Table 1). These observations collectively demonstrate that concurrent loss‐of‐function mutations in *NFU2* and *NFU3* have additive effects on the abundances of 4Fe‐4S‐containing PSI core subunits, PSI activity, PSII activity, and mutant phenotypes. This is probably because both NFU2 and NFU3 play major roles in the maturation of 4Fe‐4S‐containing PSI core subunits.

Interestingly, the magnitude of reduction in PSI activity in the *nfu2‐1* ^−/−^ *nfu3‐2* ^+/‐^ and *nfu2‐1^+/‐^ nfu3‐2* ^−/−^ sesquimutants was much more evident than the magnitude of reduction in PSII activity (Figure 2b,c). This suggests that the decline of PSII activity in these sesquimutants is likely the result of the substantial reduction in PSI activity. Consistent with this hypothesis, the abundances of PSII core subunits D1 and CP43 in the *nfu2‐1* ^−/−^ *nfu3‐2* ^+/‐^ and *nfu2‐1^+/‐^ nfu3‐2* ^−/−^ sesquimutants were statistically similar to those in the single mutants (Figure 3b; Table 1). This is in sharp contrast to the substantial reductions of 4Fe‐4S‐containing PSI core subunits PsaA, PsaB, and PsaC in the sesquimutants (Figure 3a; Table 1). Such differential reductions in PSI and PSII activities and core protein abundances had also been observed in the *nfu2* and *nfu3* single mutants (Nath et al., 2016; Touraine et al., 2004). It is established that defects in PSI or intersystem electron transport may result in secondary reductions in PSII activity (Amann et al., 2004; Lennartz et al., 2001; Walters et al., 2004).

### 4.2 NFU1 contributes to the biogenesis of 4Fe‐4S and 3Fe‐4S clusters in the chloroplast

It was proposed that NFU1 may bind and deliver iron‐sulfur clusters (i.e., 4Fe‐4S and 3Fe‐4S) to recipient apoproteins (Roland et al., 2020). Using spectroscopic analysis, we found that the affinity‐purified recombinant NFU1 protein had a broad absorption peak at ~410 nm (Figure 4b), a feature of 4Fe‐4S and 3Fe‐4S clusters (Kennedy et al., 1984; Nakamaru‐Ogiso et al., 2002). Such feature had also been observed in NFU3, a known carrier for 4Fe‐4S and 3Fe‐4S clusters in the chloroplast (Nath et al., 2016, 2017). As shown in Figure 4b, the 410‐nm broad absorption peak of the as‐purified NFU1 protein disappeared after the addition of the reducing agent sodium dithionite, which suggests that the iron‐sulfur clusters bound to NFU1 are redox‐labile (Nakamaru‐Ogiso et al., 2002). Furthermore, in vitro reconstitution experiments showed that NFU1 has an iron‐sulfur scaffold function. These experiments led to the hypothesis that NFU1 may act as a carrier in the biogenesis of 4Fe‐4S and 3Fe‐4S clusters in the chloroplast. Consistent with this hypothesis, loss‐of‐function mutations in the *NFU1* gene resulted in reductions in the levels of 4Fe‐4S‐containing PSI core subunits (e.g., PsaA and PsaC) and 3Fe‐4S‐containing FD‐GOGAT (Table 3; Figure S1). The *nfu1‐1* and *nfu1‐2* mutants displayed an average of 13% reduction in these 4Fe‐4S‐ and 3Fe‐4S‐containing chloroplastic proteins.

### 4.3 PSI is a target of NFU1 action

The *nfu1‐1* and *nfu1‐2* mutants showed an average of 15% reduction in PSI core subunits such as PsaA and PsaC (Table 3). In line with the declines in PSI core subunits, the *nfu1‐1* and *nfu1‐2* mutants displayed a ~16% reduction in PSI activity (Figure 6; Table 2). These data suggest that NFU1 contributes to the biogenesis of 4Fe‐4S clusters present in PSI core subunits and that PSI is a target of NFU1 action. Such reductions in PSI core subunit abundances and PSI activity did not result in noticeable declines in PSII activity or total chlorophyll content (Table 2), presumably due to the relatively modest magnitude of the reduction. Although immunoblot analysis and PSI activity measurements of the *nfu1* mutants (Figure 6; Tables 2 and 3) suggest that NFU1 participates in the maturation of 4Fe‐4S‐containing PSI core subunits, we should not exclude the possibility of NFU1 being involved in the maturation of other 4Fe‐4S‐containing proteins in the chloroplast. Bimolecular fluorescence complementation assays showed that NFU1 may interact with other chloroplastic 4Fe‐4S‐containing proteins (Berger et al., 2020).

### 4.4 Relative contributions of NFU1, NFU2, and NFU3 in the biogenesis of chloroplastic 4Fe‐4S clusters

The *nfu1‐1* and *nfu1‐2* mutants displayed an average of 15% reduction in the abundances of 4Fe‐4S‐containing PSI core subunits (Table 3). In this study, the *nfu2‐1* mutant displayed an average of 77% reduction in the abundances of such PSI core subunits, and the *nfu3‐2* mutant displayed an average of 88% reduction (Table 1). Similar trends in the abundances of PSI core subunits had been previously observed in the *nfu2‐1* and *nfu3* single mutants (Nath et al., 2016; Touraine et al., 2004). These results suggest that all three plastid‐targeted NFU proteins contribute to the biogenesis of 4Fe‐4S clusters in the chloroplast. Although different locations of T‐DNA insertions in a gene may result in phenotypic variations, mutation severity is generally a good indicator of the relative importance of the gene product. The relative magnitudes of reductions in 4Fe‐4S‐containing PSI core proteins in the loss‐of‐function mutants are consistent with the hypothesis that NFU3 contributes more than NFU2 and NFU2 contributes more than NFU1. In line with the presumed contributions of the three plastid‐targeted NFU proteins, the *nfu3‐2*, *nfu2‐1*, and *nfu1* single mutants displayed 86%, 78%, and 16% reduction in PSI activity, respectively (Figure 2b; Table 2). The relative contributions of three plastid‐targeted NFU proteins in the biogenesis of chloroplastic 4Fe‐4S clusters could reflect their protein expression levels in photosynthetic tissues. According to immunoblot analysis, the three plastid‐targeted NFU proteins are differentially expressed in different tissues (Touraine et al., 2019; Yabe et al., 2004). For example, the NFU2 protein is expressed in true leaves, cauline leaves, flower stalks, flowers, green siliques, and roots (Touraine et al., 2019; Yabe et al., 2004). The unique expression of NFU2 in the roots led to the discovery that NFU2 participates in the maturation of 2Fe‐2S‐containing dihydroxyacid dehydratase and is required for the synthesis of branched‐chain amino acids in Arabidopsis roots (Touraine et al., 2019). Unlike NFU2 or NFU3, the NFU1 protein appeared to be expressed at a very low level in leaves (Yabe et al., 2004).

## 5 CONCLUSIONS

As discussed above, concurrent loss‐of‐function mutations in the *NFU2* and *NFU3* genes have additive effects on the declines of 4Fe‐4S‐containing PSI core subunits. Thus, the *nfu2‐1* ^−/−^ *nfu3‐2* ^+/‐^ and *nfu2‐1^+/‐^ nfu3‐2* ^−/−^ sesquimutants had much lower PSI and PSII activities, much less chlorophyll, and a much smaller plant size than the *nfu2‐1* and *nfu3‐2* single mutants. These observations also suggest that NFU2 and NFU3 play major roles in the biogenesis of chloroplastic iron‐sulfur clusters. Spectroscopic and in vitro reconstitution experiments of the recombinant NFU1 protein led to the hypothesis that NFU1 may act as a carrier in the biogenesis of 4Fe‐4S and 3Fe‐4S clusters in the chloroplast. Consistent with this hypothesis, the loss‐of‐function *nfu1* mutants displayed significant reductions in the abundances of 4Fe‐4S‐containing PSI core proteins and 3Fe‐4S‐containing FD‐GOGAT. The significant declines of PSI activity and 4Fe‐4S‐containing PSI core subunits in the *nfu1* mutants suggest that PSI is a main target of NFU1. Results in this and previous studies showed that all three plastid‐targeted NFU proteins contribute to the biogenesis of 4Fe‐4S clusters in the Arabidopsis chloroplast. The relative magnitudes of reductions in 4Fe‐4S‐containing PSI core proteins in the loss‐of‐function mutants are in line with the hypothesis that NFU3 contributes more than NFU2 and NFU2 contributes more than NFU1. The relative contributions of NFU3, NFU2, and NFU1 to chloroplastic 4Fe‐4S biogenesis are consistent with the plant size, chlorophyll content, PSI activity, and PSI activity of the corresponding loss‐of‐function mutants.

## ACCESSION NUMBERS

Sequence data of related genes/proteins can be found in the GenBank/EMBL databases under the following accession numbers: *N* *FU1*, At4g01940; *NFU2*, At5g49940; *NFU3*, At4g25910; and *ACT2*, At3g18780.

## CONFLICTS OF INTERESTS

The authors declare no conflict of interest.

## AUTHOR CONTRIBUTIONS

M.B.S. and Jun Zhao performed most of the experiments and analyzed most of the data; Jessica Zhang performed some experiments; F.Y. edited the article; Y.L. conceived the project, conducted some specific experiments, analyzed the corresponding data, and wrote and edited the article.

## Supporting information

Supplementary Material

## Data Availability

All relevant data can be found within the manuscript and its supporting materials.
