# Supplementary material for: Functional relationships of three NFU proteins in the biogenesis of chloroplastic iron‐sulfur clusters
Source: Plant Direct. 2021 Feb 2;5(1):e00303. doi: 10.1002/pld3.303 (PMC7851846; doi:10.1002/pld3.303)
Supplement: Supplementary file 1 — Supplementary Material [file PLD3-5-e00303-s001.pdf]

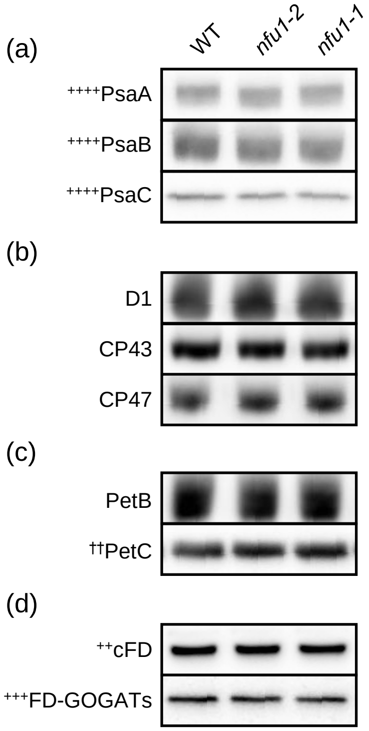

Figure S1. Immunoblots of representative iron-sulfur cluster-containing proteins and other photosynthetic proteins in the wild type and *nfu1* mutants. (a) Immunoblots of PSI reaction-center proteins PsaA, PsaB, and PsaC. (b) Immunoblots of PSII reaction-center proteins D1, CP43 and CP47. (c) Immunoblots of cytochrome b_6f complex proteins PetB and PetC. (d) Immunoblots of chloroplast ferredoxin cFD and ferredoxin-dependent glutamine oxoglutarate aminotransferases FD-GOGATs. Thylakoid membrane proteins were used in (a-c) and were loaded on an equal chlorophyll basis. Leaf total proteins were used in (d) and were loaded on an equal total protein basis. Symbols ++, ††, +++, and ++++ indicate that the protein binds to classic 2Fe-2S, Rieske-type 2Fe-2S, 3Fe-4S, and 4Fe-4S, respectively. Four-week-old plants were used for SDS-PAGE and immunoblot analysis in this figure.

Table S1. Primers used in this study

Primer	Sequence	Use
SALK_039254LP	5'-TTCGTCCCAGTACTCAAATGG-3'	Left genomic primer for *nfu2-1*
SALK_039254RP	5'-AATGGTTTCTGGTCCCATCTC-3'	Right genomic primer for *nfu2-1*
GABI_791C01LP	5'-CCAAACTGGACTCTTGACCAG-3'	Left genomic primer for *nfu3-2*
GABI_791C01RP	5'-ATCAATTTCAAAACCAAGGGG-3'	Right genomic primer for *nfu3-2*
GABI_661F04LP	5'-TGAATACAAACATGAATGGCC-3'	Left genomic primer for *nfu1-1*
GABI_661F04RP	5'-GTCGAACGAAATCAGCAGAAC-3'	Right genomic primer for *nfu1-1*
SALK_038073LP	5'-TGAATACAAACATGAATGGCC-3'	Left genomic primer for *nfu1-2*
SALK_038073RP	5'-GTCGAACGAAATCAGCAGAAC-3'	Right genomic primer for *nfu1-2*
LBa1	5'-TGGTTCACGTAGTGGGCCATCG-3'	Left border primer for SALK lines
o8409	5'-ATATTGACCATCATACTCATTGC-3'	Left border primer for GABI lines
NFU1_L	5'-TTGATTTGGTGCTTGAGGAT-3'	Left qRT-PCR primer for *NFU1*
NFU1_R	5'-CCCATTGTCATAGTTGTTGAAG-3'	Right qRT-PCR primer for *NFU1*
ACT2_L	5'-CAAAGGCCAACAGAGAGAAGA-3'	Left qRT-PCR primer for *ACT2*
ACT2_R	5'-ATCACCAGAATCCAGCACAA-3'	Right qRT-PCR primer for *ACT2*
NFU1_BamH1_ATG	5'-ACACAGGATCCATGATGGCTTCTCTCGCGAC-3'	Forward cloning primer for *NFU1*
NFU1_BamH1_noTP	5'-ACACAGGATCCGCATCTGGTGTATCTTCAGGT-3'	Forward cloning primer for *NFU1*
NFU1_Xho1_TAG	5'-ACACACTCGAGCTAGCTTGTAAAGGTTACATTAG-3'	Reverse cloning primer for *NFU1*
